# Dissecting the forces that shape crops: an interview with Ryohei Terauchi

**DOI:** 10.1186/s12915-018-0598-0

**Published:** 2018-11-01

**Authors:** Ryohei Terauchi

**Affiliations:** 1 Laboratory of Crop Evolution, Graduate School of Agriculture, Kyoto University, Kyoto, Japan; 2 Iwate Biotechnology Research Centre, Kyoto, Japan

**Keywords:** Yam, Crops, Evolution, Plant pathogens, Genome

## Abstract

Ryohei Terauchi is a Professor at Kyoto University and a Group Leader at the Iwate Biotechnology Research Center, Japan, studying the evolution of crops and their pathogens. In this interview, Ryohei describes his research interests, how the revolution in sequencing technology helped improve our understanding of orphan crops, and who are the scientists that inspire him.

## What are the questions driving your research?

How has evolution shaped such a diversity of species on earth? I am particularly interested in co-evolutionary processes of species caused by their interactions. My major questions include how plant–human interactions have resulted in the domestication of various crops and how crop–pathogen interactions are shaping their current coevolution. I wish to address these questions from genomics perspectives.

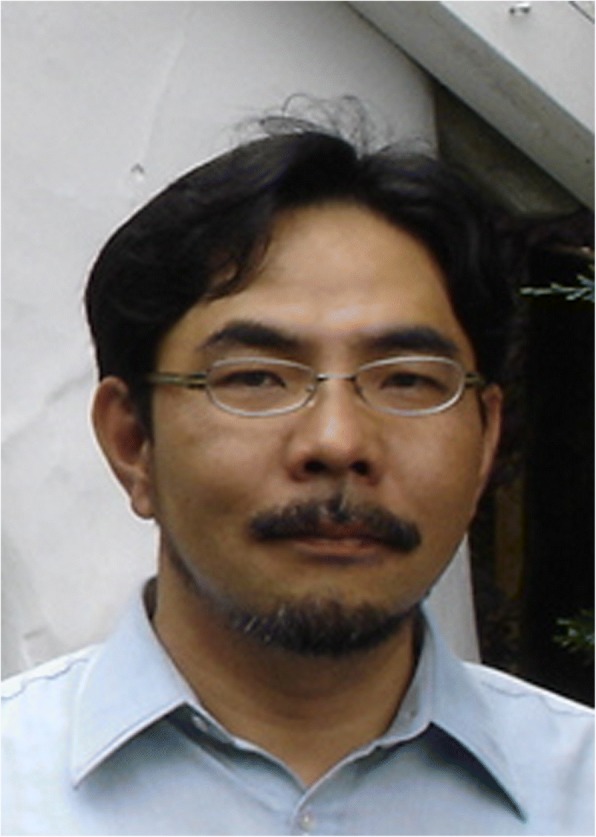



## Your recent paper in *BMC Biology* described the genome sequence of the white Guinea yam, a staple crop of the African continent. Can you tell us a little bit about this study?

Yam crops belonging to the genus *Dioscorea* are an important staple food in tropical and subtropical areas of the world. However, research efforts on it have been very limited. We are proud of our paper on the genome sequence of Guinea yam published in *BMC Biology* in 2017 [1], which shows that the genomics revolution, ushered by the development of DNA sequencing technology, provides useful information for the improvement of orphan crops, so far neglected but regionally very important crops, including yams.

## Looking back, is there a project that your lab pursued that stands out for you as particularly inspiring, tough, or simply memorable?

A paper by Yoshida et al. [2] reporting the isolation of three avirulence genes from the rice blast pathogen *Magnaporthe oryzae* by whole genome sequencing has determined the direction of our genomics research. I also consider the papers by Abe et al. [3] and Takagi et al. [4] important as these have enabled us to quickly link phenotypes and genotypes, which is instrumental for understanding evolution.

## Is there a paper or a scientist that inspired you, or was seminal for your research?

I admire the population geneticist JBS Haldane with his deep insights into evolution, advocacy of quantitative analysis of biological phenomena, and a non-conforming style in science and life. I was personally very much inspired by my mentor Guenter Kahl, who taught me the importance of freedom and independence in science and life. I am very happy to name Sophien Kamoun, who has always inspired us with his great insights into science and openness during our long-time collaboration.

## What are your guiding principles for running a lab? Do you have any advice to share with our readers?

Only independent and free thinking will result in innovative ideas and findings.

## If you could, what would you tell your younger self?

Spend more time reading classic pieces that have stood the test of time.

**Website:** http://www.crop-evolution.kais.kyoto-u.ac.jp/?lang=en, http://genome-e.ibrc.or.jp/home

**Twitter:** @ryoheiterauchi
